# Cases Reported for the Atlanta Medical & Surgical Journal

**Published:** 1866-05

**Authors:** J. T. Banks

**Affiliations:** Griffin


ATLANTA

Medical and Surgical Journal.

NEW SERIES.

Vol. VII. MAY, 1866. No. 3.

### ORIGINAL COMMUNICATIONS.

### ARTICLE I.

*Cases Reported for the Atlanta Medical & Surgical Journal.*—By Dr. J. T. Banks.

Case No 1.—John Davis, a powerful, but dissipated soldier of the C. S. A., and permanently lame from injuries in the late war, received several wounds by a pointed dagger, between three and four o'clock, on the morning of December 6th, 1865, while on a drinking spree, in an eating saloon of this city. I was called to see him about five o'clock, and found him romoved about three-fourths of a mile, to which place he had been carried in a buggy. On examination, I found three punctured wounds, made by a pointed dagger with sharp edges, but only *one* of a serious character, which was a penetrating wound, in the left side, about midway between the crest of ilium and the floating ribs. It was about three-fourths of an inch in extent, and opened directly towards the centre of the abdominal cavity. On inquiring, I learned that Dr. ——— had seen and examined the wound, and gave it as his opinion that it did not penetrate the cavity; but from the amount of collapse, little loss of blood, and severe pain complained of, and located in the umbilical region, I was satisfied that the dagger had been plunged deep into the cavity, but could not diagnose any special injury within. His pulse was extremely feeble—at times imperceptible. I dressed his wounds with cold water dressing, and applied the same over abdomen. Gave him one gr. of sulp. morphia in ℥ ii of whisky, and left two more gr. doses to be given every three or four hours until the severe pain subsided; also ordered perfect quiet, with the cautious use of stimulants until re-action was established. December 5.—Condition no better. Re-action imperfect; partial coma, supposed to be the result of doubling the amount of morphia prescribed, but when aroused, complained of the same pain. Bowels distended since first visit; passed urine; pulse extremely feeble; prescribed warm brandy toddy, and constant application of cold water over abdomen.

He continued to sink, and died at four o'clock next morning.

Post mortem examination, thirty hours after death, assisted by Dr. N. B. Drewry.

Only one wound penetrated any cavity of body. On opening abdominal cavity, we found a very large amount of dark, semi-coagulated blood, filling up every space among the convolutions of small intestines on left side. To find the source of so much hemorrhage, we carefully removed about two large washpansful of this dark, semi-coagulated blood, when I carefully traced the rout of dagger beneath the descending colon, just above the sigmoid flexure, and beneath the small intestines, to the *abdominal aorta*, which I found punctured about two inches above its bifurcation, the puncture being about one-fourth of an inch in extent. His death was caused by hemorrhage from this puncture.

The professional interest in this case, is the length of time he lived after such a puncture of the aorta; and the vast amount of internal hemorrhage, which could not have been less than 150 oz.; teaching how low the amount of the circulating mass may be reduced without inducing syncope, when the recumbent posture is maintained, and the blood drawn off in a small stream.

It is to be regretted that a correct diagnosis could not be made soon after the reception of the wound, thereby losing a favorable opportunity of ligating the abdominal aorta in a healthy subject.

Case No. 2.—In connection with Dr. M. J. Daniel, on the morning of ———, 1865, I was called to see Colonel R., who had been severely injured in a railroad accident the over night. On my arrival I found him in the care of Dr. Smith, of Athens, who was on the train at the time of the accident, and had applied such temporary dressing as the case demanded, while kindly remaining with him (eight or ten hours) until other medical aid could be obtained.

We found him in a state of extreme collapse. Skin cold and bathed in a profuse perspiration; pulse rapid, feeble, and often imperceptible; not much complaint of pain in quietude, but extreme nausea and vomiting. On examination, I found a compound fracture of left leg, both bones broken in upper part of *middle third.* Also, a fracture of thigh at juncture of lower and middle third, with all the intermediate soft parts very much contused—so much so around the knee-joint, that a correct and satisfactory diagnosis of its condition could not be made out, until several days after, when I punctured a large fluctuating tumor, on the inside of the knee, and discharged five or six oz. of dark, thin, disorganized blood, after which I had no difficulty in deciding upon the non-implication of the knee-joint in the injury. The amount of contusion and effusion around the joint, with distinct fluctuation, led to the belief that a serious lesion of the joint existed, and that the fluid, giving the feeling of fluctuation, was in the synovial sac. Fortunately, the joint escaped injury, and the fluctuation was caused by hemorrhage into the surrounding cellular tissue. There were two wounds of the leg communicating with the fracture, one anterior with the tibia, and the other posterior and lower down, with the fibula. In addition to these there was a dislocation of the right sacro-iliac symphysis, caused by a severe blow on the tuberosity of ischium, driving the ilium upwards and backwards half an inch or more. The same blow inflicted some injury on urethra. He had several contusions over other parts of the body, but of little consequence. His condition was esteemed critical; life was threatened with extinction every hour; nine hours had passed, not only without any indication of re-action, but with a marked decline of vital energy in last hour; a death-like paleness covered his features; he was bathed in dissolution's clammy damp, with only caloric enough left to prevent the congelation of the fountain of life. Under the circumstances, we determined to make him as comfortable as possible without any mechanical appliances, and address our treatment to his restoration, believing that his chances for recovery were not more than five in a hundred. We prescribed morphia, hot brandy and whisky punches, hot coffee, highly seasoned soups, camphor juleps, with carb. ammonia; dry heat to feet and legs; mustard over stomach, &c., for twenty-four hours, with little or no improvement, his stomach rejecting everything. We then gave him champagne and ice—stopped all allowance of water, except so much as he obtained from ice slowly melting in his mouth and stomach. Under this plan of treatment, with the aid of morphia, he so far re-acted in the next twenty-four hours, as to justify a consultation on the propriety of amputation of left leg. In this, three other medical friends of our city kindly gave us their opinion, and all concurred in the decision that amputation would be absolutely necessary when he re-acted sufficiently. It was then fifty-eight or sixty hours after the accident. This sad information being communicated, I left him to his reflections until a more permanent re-action could be obtained, his condition to be watched every hour. About three hours after, Dr. Daniel informed me that Colonel R. had expressed desire for Dr. W. F. Westmoreland to be called in. Knowing that Dr. W. could not see him in less than twenty hours, I gave it as my opinion that the time to operate in safety would pass before he could get there. After an examination on the next day, by Dr. W., he gave it as his opinion that the time for amputation had passed, if, indeed, he had ever been able to undergo it—the local excitement having set up before his system had fully re-acted. We then decided to give him the chance of life and limb together, (Colonel R. had *apriori* made the same decision,) and directed our treatment to his comfort and ease, both local and general. For two weeks or more, the war between life and death was fierce and fearful, and I am constrained to say, that had he been less indifferent to life, (being not fearful of death or its consequences,) or had he been of a despondent temperament, without a fixed purpose to live for, and a determination to live to accomplish it, death would have triumphed; but, aided by his indomitable will, we have succeeded in setting him afloat, aided by two additional supports to locomotion, hinged in his axillæ.

The treatment after re-action had been established, was *position* for broken limb; no effort at reduction of sacro-iliac symphysis. At the end of the sixth week, there was a firm osseous union of fractured femur, but free motion in fracture of leg. Applied starch bandage, and in a short time had it rewarded by a firm union.

The dislocation of sacro-iliac symphysis, was followed by almost complete paralysis and anæsthesia of right leg for two or three weeks, but any motion of right hip was attended by severe pain. Strength of right limb slowly improving; left leg two inches *short* and of very little *use*.

GRIFFIN, March 21st, 1866.

